# Methodological considerations for a randomised controlled trial of podiatry care in rheumatoid arthritis: lessons from an exploratory trial

**DOI:** 10.1186/1471-2474-8-109

**Published:** 2007-11-06

**Authors:** Deborah E Turner, Philip S Helliwell, James Woodburn

**Affiliations:** 1 HealthQWest, School of Health & Social Care, Glasgow Caledonian University, Glasgow, UK; 2 Academic Unit of Musculoskeletal Disease, School of Medicine, University of Leeds, Leeds, UK

## Abstract

**Background:**

Whilst evidence exists to support the use of single treatments such as orthoses and footwear, the effectiveness of podiatry-led care as a complex intervention for patients with rheumatoid arthritis (RA) related foot problems is unknown. The aim of this study was to undertake an exploratory randomised controlled parallel arm clinical trial (RheumAFooT) to inform the design and implementation of a definitive trial and to understand the potential benefits of this care.

**Methods:**

Patients with a definite diagnosis of RA, stable drug management 3 months prior to entry, and a current history of foot problems (pain, deformity, stiffness, skin or nail lesions, or footwear problems) were recruited from a hospital outpatient rheumatology clinic and randomised to receive 12 months of podiatry treatment or no care. The primary outcome was change in foot health status using the impairment/footwear (LFIS_IF_) and activity limitation/participation restriction (LFIS_AP_) subscales of the Leeds Foot Impact Scale. Disease Activity Score (DAS), Health Assessment Questionnaire (HAQ) score and walking speed (m/s) were also recorded.

**Results:**

Of the 80 patients identified, 64 patients were eligible to participate in the pilot and 34 were recruited. 16 patients were randomised to receive podiatry led foot care and 18 received no care. Against a backdrop of stable disease (DAS and HAQ scores), there was a statistically significant between group difference in the change in foot health status for foot impairment (LFIS_IF_) but not activity/participation (LFIS_AP_) or function (walking speed) over 12 months. In the podiatry arm, 1 patient declined treatment following randomisation (did not want additional hospital visits) and 3 self-withdrew (lost to follow-up). Patients received an average of 3 consultations for assessment and treatment comprising routine care for skin and nail lesions (n = 3), foot orthoses (n = 9), footwear referral to the orthotist (n = 5), and ultrasound guided intra-articular steroid injection (n = 1).

**Conclusion:**

In this exploratory trial patients were difficult to recruit (stable drug management and co-morbid disease) and retain (lack of benefit/additional treatment burden) but overall the intervention was safe (no adverse reactions). Twelve months of podiatry care maintained but did not improve foot health status. These observations are important for the design and implementation of a definitive randomised controlled trial.

**Trial Registration:**

ISRCTN: 01982076

## Background

Rheumatoid arthritis (RA) is a long-term inflammatory disease affecting about 1% of the population and is characterised by joint inflammation, progressive joint destruction and increasing functional disability [1]. Cross sectional studies in patients with established disease suggest that foot problems occur in around 80–90% of patients typically in the form of forefoot pain, stiffness, deformity, skin pressure lesions such as callosities and problems related to footwear fit and comfort [2,3]. In early disease (typically < 2 years), active joint disease leading to foot pain and altered joint function may be problematic in about one-third of patients [4,5]. Overall, the burden of foot disease in RA is substantial and impacts negatively on health-related quality of life [4,6].

Treatment for RA aims to decrease and control pain and stiffness, reduce or prevent cumulative joint damage, maximise physical function, and improve quality of life. Podiatrists are regarded as the expert on foot problems and are well placed to assess, advise and treat patients with RA [7]. Expert opinion and clinical practice guidelines suggest patients should be treated early in the disease with an emphasis on treating biomechanical problems using customised foot orthoses [7,8]. In established disease, typical podiatry interventions such as orthoses, specialist footwear, and debridement of callus aim to lessen foot pain and improve function [9-11]. However, podiatry services in UK rheumatology centres are scarce despite significant unmet demand [12,13]. Indeed, a recent survey indicated that unmet demand for chiropody or footwear was reported in almost half (46%) of RA patients, and this ranked the highest amongst requested services [13].

A recent systematic review suggests that RA patients are likely to benefit from foot orthoses and specialist footwear with little or no evidence of harm [14]. In contrast, scalpel debridement for painful plantar callosities provides only short term pain relief for approximately 7 days. The benefits and harm of foot surgery have not been established as no randomised controlled trials (RCTs) have been conducted [14]. In routine clinical practice the podiatrist may play an important role in the larger and more complex delivery of foot care for patients with RA [15,2]. The Medical Research Council (MRC) framework for evaluating complex interventions recommends a phased approach through a continuum of increasing evidence [17]. Since much of the pre-clinical work has already been reported and components of podiatry intervention are relatively well defined, the aim of this study was to conduct an exploratory (phase II) RCT (RheumAFooT – Rheumatoid Arthritis Foot Trial) of podiatry-led foot care in RA patients with foot problems. The trial was designed to answer 2 specific questions; (1) what are the key methodological features for a pragmatic RCT to evaluate the effectiveness of podiatry-led foot care in RA patients with foot problems, and (2) how effective is this intervention?

## Methods

### Ethical approval

Leeds (West) Local Research Ethics Committee, Leeds UK (03/078) provided the ethical review and approval.

### Patient involvement

A focus group was conducted with patients who had previously received podiatry care. Areas explored included their expectations and experiences of podiatry care in the local setting, as well as opinion on the mode and delivery of care, those providing care and alternative experiences including medical management, surgery and self-care. Specific input was sought for the study design and ethical implications, patient recruitment and involvement, and development and use of study materials including consent forms and information leaflets. Emerging themes and key issues were identified, documented and summarised in a debriefing session and the relevant findings incorporated into the phase II exploratory trial.

### Participants

Participants were recruited to the study from the medical and nurse-led rheumatology outpatient clinics at a single secondary care centre (Leeds General Infirmary, Leeds, UK). Patients were identified from new referrals to the hospital podiatry service by clinicians. Patients were eligible for inclusion if they had a definite diagnosis of RA (satisfying the 1987 American Rheumatism Association revised criteria for RA [18]), were between 18–80 years of age, were able to read and write English and had a current history of foot problems with an impairment/footwear subscale (LFIS_IF_) score of ≥4 points on the Leeds Foot Impact Scale [19]. Patients were also required to have stable drug management in the 3 months prior to recruitment. Patients were excluded if they had received podiatry treatment within the last 3 months, had foot problems primarily related to other medical conditions such as diabetes mellitus and peripheral vascular disease, or had significant complications with their RA placing them at risk of foot ulceration or infection, especially those on biologic and other immunosuppressant therapy.

### Randomisation

Random allocation was performed by the Central Randomisation Service based at the Northern and Yorkshire Clinical Trials and Research Unit. The allocation was conducted independently of the researcher undertaking the outcome assessments. Consultations for those patients randomised to receive podiatry were undertaken by clinicians blinded to the patient's inclusion in this study and patients were also instructed not to inform clinicians of their involvement.

### Interventions

The podiatry interventions were undertaken by a small team of podiatry staff working in rheumatology outpatients. The podiatrists were blinded to the patient's allocation to the intervention arm. Accordingly, the trial was a pragmatic evaluation of current care where patients were assessed, treated and followed-up according to need. Pre-clinical work showed that the podiatrists delivered individualised care comprising one or more treatments: foot health advice including disease information, treatment guidance and self-management strategies; prescription footwear therapy and foot orthotic interventions including customised and non-customised devices; palliative or surgical treatments for nail disorders; palliative care for non-ulcerated pressure-induced skin lesions including corns and callosities; and maintenance of optimal tissue viability for pre-ulcerative lesions and wound care management for ulcerative skin lesions. In some cases referrals were made to other diagnostic services and specialists including the rheumatologist, rheumatology nurse practitioner, orthopaedic surgeon, physiotherapist, occupational therapist and orthotist. In this unit one podiatrist working in a specialist role was trained to perform intra-articular corticosteroid injections. Patients randomised to the non-intervention arm did not receive foot care for the 12 month study period. Usual outpatient medical/nurse-led care was maintained for both patient groups.

### Outcomes

The primary outcome was the LFIS_IF _subscale of the Leeds Foot Impact Scale [19]. This was measured at 8, 4 and 0 weeks prior to treatment to establish a precise baseline measure and repeated at 3, 6, 9 and 12 months following the start of podiatry care. Secondary outcomes included the activity limitation/participation restriction subscale of the LFIS (LFIS_AP_), the Disease Activity Score (DAS) using 28 joint counts, the Stanford Health Assessment Questionnaire score (HAQ) for functional disability, and walking speed (a quantitative measure of global function). Demographics, disease characteristics and drug use were also recorded at baseline. The type and frequency of podiatry care along with adverse effects were also monitored in the podiatry intervention group.

### Data analysis

The outcome data was analysed using SPSS 13.0 for Windows. The main study outcomes were summarised as mean (SD) or median (interquartile range) depending on the distribution of the data. The preliminary evaluation of the relative effectiveness of the podiatry intervention was determined by comparing the change in primary outcome between baseline and 12 months. Those patients who did not receive the allocated intervention or were lost to follow-up were invited to receive an evaluation at 12 months. If they declined their last available data was used for analysis. The analysis was based on the Hodges-Lehman estimate of the median difference with 95% confidence interval based on a two-sided Mann-Whitney U-test.

## Results

### Patient involvement

Findings from the focus group showed that none of the patients had received information about the role of the podiatrist prior to referral. Expectations were described as low, neutral, hesitant or hopeful. Patients expected to receive nail care, treatment for hard skin or insoles based on their understanding of chiropody treatment. Three-quarters of patients had found their treatment helpful. Those dissatisfied cited repeat use of ineffective treatments such as insoles and footwear and delay of other subsequently more effective treatments such as joint injections and orthopaedic foot surgery. Use of a non-intervention arm was universally supported when presented among other scenarios and 12 months was considered the optimum time to evaluate the effects of treatment. Some patients were aware that podiatry services were not widely available in rheumatology clinics. Patients highlighted potential recruitment and follow-up problems, especially for patients in work, due to difficulties attending clinic for additional non-medical treatments, especially when expectations were low. The patients contributed to the successful development of trial information including, for example, the patient information leaflet.

### Trial Profile

**Figure 1:**
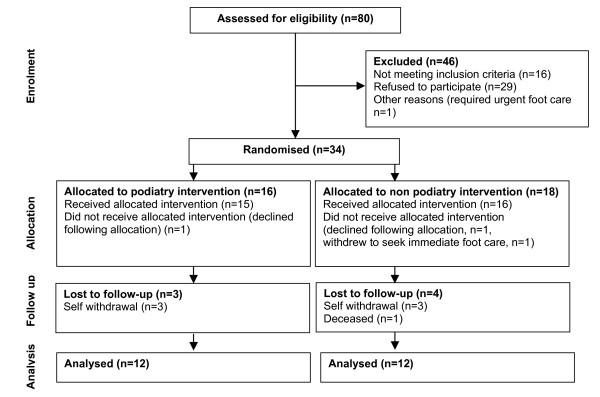
Trial profile and participant flow.

Eighty patients attending rheumatology outpatient clinics were identified as potential participants. Twenty-nine patients refused to participate, 6 patients did not meet the inclusion criteria and one patient who required urgent treatment for a foot ulcer was ineligible (Figure 1). Thirty-four patients were randomly allocated to the podiatry intervention (n = 16) and no podiatry intervention (n = 18) groups. In the podiatry group, 15 patients received the allocated treatment and one declined immediately following allocation. In the no podiatry intervention group, 16 patients received the allocated intervention, 1 patient declined immediately following allocation and 1 patient withdrew. Numbers lost to follow up were 3 for the podiatry and 4 for the no podiatry intervention groups.

### Demographic and clinical characteristics

The baseline characteristics of the patients by allocation are shown in Table 1. Patients in the podiatry intervention arm were younger and had a shorter disease duration, lower disease activity and less functional disability but differed significantly only in terms of age (p = 0.03). Patients were well matched for pharmacological management profiles. Foot pain on the LFIS subscale was similar but the podiatry group experienced less foot-related disability and walked faster although none of these baseline scores were significantly different.

**Table 1:** Baseline characteristics of patients by allocation

**Demographics and disease characteristics**	**No intervention**	**Podiatry**
N	18	16
Female: n (%)	13 (72%)	12 (75%)
Age: years (SD)	64.6 (12.1)	56.8 (10.2)
Disease duration: years (SD)	16.7 (10.1)	14.1 (11.9)
DAS (0–10): median score (IQR)	4.3 (3.5,5.2)	3.7 (3.1,4.2)
HAQ score (0–3): median score (IQR)	1.04 (0.48,1.71)	0.75 (0.63,1.13)
BMI: median score (IQR)	27.0 (22.6,32.3)	27.0 (24.8,30.5)

**Pharmacological management**		

Analgesics: n (%)	8 (44%)	6 (38%)
NSAIDs: n (%)	9 (50%)	8 (50%)
DMARDs: n (%)	12 (67%)	14 (88%)
Biologic agent: n (%)	2 (11%)	1 (6%)
Corticosteroid: n (%)	5 (28%)	4 (25%)
Other: n (%)	2 (11%)	2 (13%)

**Foot disease**		

LFIS_IF _(0–21): median score (IQR)	13 (12,14)	15 (12,16)
LFIS_AP _(0–30) median score (IQR)	21 (16,26)	17 (15,24)

**Function**		

Walking speed (m/s): median score (IQR)	0.70 (0.40,1.09)	0.88 (0.76,1.00)

DAS, Disease Activity Score; HAQ, Health Assessment Questionnaire; BMI, Body Mass Index; NSAIDs, Non-Steroidal Anti Inflammatory Drugs; DMARDs, Disease Modifying Anti-Rheumatic Drugs, LFIS, Leeds Foot Impairment Score (IF, Impairment/footwear subscale, AP, Activity limitation/participation restriction subscale). Values are presented as mean (standard deviation) or median (inter-quartile range).

### Primary outcome

There was a statistically significant between-group difference in the primary outcome (Table 2). However there was no change from the baseline score in the podiatry intervention group and a small deterioration in foot impairment in the no podiatry group. Overall the effect size was small and clinically unimportant.

**Table 2:** Change in primary and secondary outcomes between baseline and 12 months.

	**Podiatry care (n = 16)**	**No podiatry care (n = 18)**		
			
**Outcome**	**Change BL-12M**	**Change BL-12M**	Difference (95% CI)*	***p *value**
LFIS_IF _(0–21)	0 (-3,1)	1 (0,3)	2 (0 to 4)	0.035
LFIS_AP _(0–30)	0 (-4,2)	0 (-2,1)	0 (-2 to 3)	0.971
DAS (0–10)	0.2 (0,0.5)	0 (0,0.8)	0 (-0.4 to 0.4)	0.791
HAQ (0–3)	0 (-0.03,0)	0 (0,0.25)	0 (-0.12 to 0.33)	0.303
				
Walking speed (m/s)	0.01 (-0.01,0.07)	-0.02 (-0.20,0.02)	0 (-0.1 to 0)	0.411

LFIS, Leeds Foot Impairment Score (IF, Impairment/footwear subscale, AP, Activity limitation/participation restriction subscale); DAS, Disease Activity Score; HAQ, Health Assessment Questionnaire; BL-12M, change in score between baseline and 12 months; CI, confidence interval. Values for the change in score from BL-12M are presented as the median (inter-quartile range). The between group difference in change scores was based on the Hodges-Lehman estimate of the median difference with 95% confidence interval based on a two-sided Mann-Whitney U-test.

### Secondary outcomes

The LFIS_AP _scores showed no change in both groups over the 12 month study period (Table 2). Over the course of 12 months patients in both arms of the trial had stable disease and no deterioration in functional disability as evidenced by the DAS and HAQ scores respectively. Similarly, walking speed was unchanged and no between group differences were detected.

### Podiatry treatment

The podiatry treatment records were not available for 1 patient. Three patients were assessed but no treatment provided within the 12 months. Of these one was referred back to the clinical nurse specialist in disease flare and a second referred directly to the orthotist for orthoses and footwear and to primary care for basic podiatry foot care. Five patients received custom orthoses from the podiatrist and were referred to the orthotist for specialist footwear. Of these, 3/5 received one additional care episode comprising ultrasound-guided intra-articular corticosteroid injection to the talonavicular joint, callus debridement, and in-growing toenail care. Six patients received either a simple insole (n = 1) or a custom orthosis (n = 5) as a single intervention. There was no evidence of any adverse reactions or side-effects to any of the interventions provided. Only 3 received routine follow-up. Of the 9 patients treated more than once in the 12 month study period only 1 had continuous care.

## Discussion

We undertook RheumAFooT as an exploratory phase II trial of podiatry care for patients with RA. Important information was gathered to inform the design and implementation of a definitive RCT. By controlling for potentially significant confounders on the main outcome, particularly pharmacological management and co-morbidity, we encountered recruitment problems. The pragmatic design revealed important problems for, (1) defining the start of treatment, (2) variation in delivery of treatment and (3) use of other medical and allied health services. However, involving a patient focus group beneficially facilitated the design and implementation of the trial especially with regards to incorporating a non-intervention arm. Acknowledging the limitations of an exploratory trial, the findings suggest that podiatry-led foot care as a complex intervention maintains but does not improve foot related impairment or disability in patients with stable controlled disease. However, no treatment leads to a small deterioration in foot health related to impairment but not disability.

There were two main challenges to recruitment in this study-stable drug management and co-morbid disease. As with all rehabilitation strategies in RA, non-pharmacological interventions are adjunct to drug management. With such powerful effects on symptoms and disease activity such combinations pose a challenge for outcome evaluation, especially for a disease with natural variation in its presentation over months and years. On reflection, the criteria set here for no change in either drug type or dosage was too stringent. Moreover, choice of medication or dosage may be changed for reasons other than controlling the disease process-tolerance and side-effects for example. Additionally, the study was based in an outpatient clinic attached to an academic unit and as such close monitoring of patients may have led to more frequent intervention. For a definitive trial it would be important to gauge the level of drug monitoring and intervention in other secondary and primary care settings to help set more pragmatic exclusion rules. Alternatively the interaction with between pharmacological and non-pharmacological treatments and changes in disease severity could be handled by statistical analyses or minimised between groups although these are difficult to anticipate. How this problem is handled in other non-pharmacological trials in RA is not well documented.

Co-morbid disease excluded one fifth of patients approached for this study, the main reasons being diabetes mellitus and peripheral vascular disease. However, exclusion was not based on diagnosis alone and was limited to those who were at risk from developing foot-related complications, primarily infection and foot ulceration. In reality most of these patients were excluded as they were in receipt of foot care or assessment/advice in other specialist clinics such as the hospital diabetic foot clinic. Exclusion by co-morbid disease requires more precise definition in future trials and should look beyond diagnosis and towards related complications and secondary treatment.

We found that over one-third of patients refused to participate in the trial. Our main concern was the use of a non-intervention arm. However, additional visits to clinic (as raised in the patient focus group), lack of perceived understanding of role and the benefit/risks of podiatry care were the three most common reasons for refusal. Indeed only one patient declined following allocation to the non-intervention group to seek care and this was matched by one patient in the intervention arm who did not want additional hospital visits. Organisations such as the Arthritis Research Campaign provide leaflets specifically detailing foot problems and their treatment but availability in outpatient clinics and uptake is not known. These findings indicate that in a definitive trial, like any new or novel treatment, the potential benefits and hazards of podiatry-led foot care must be fully explained during the recruitment of patients before they make an informed choice. Throughout the trial we were encouraged that loss to follow up was comparable between groups with no strong evidence of resentful demoralisation (deliberate withdrawal of patients in the control group who perceive differential benefit for the intervention group).

The findings suggest that a non-treatment parallel arm can also be used in a definitive trial. However, other approaches such as a patient preference design or unequal randomisation, e.g., 2:1 in favour of receiving the intervention, could improve recruitment [20]. Alternatives, such as a minimal or sham intervention arm, for example using an information leaflet or sham insole, could also be considered. However, sham procedures are not suitable within pragmatic trials as they do not model usual practice. Furthermore, sham physical agents mimicking interventions such as scalpel debridement and foot orthotics may have physiological effects that are therapeutic [11]. Indeed, we have encountered this problem in a previous trial and do not favour this approach [11].

In this exploratory trial the groups were well balanced at baseline for all but one demographic or disease variable indicating that the automated randomisation worked and was feasible for this type of study. In a definitive trial, we suggest the use of minimisation – where patients are allocated to a particular treatment depending on the characteristics of those participants already enrolled. Minimisation ensures excellent balance across important prognostic factors and is recommended for small trials [21]. Factors to minimise imbalance should include age, gender, disease duration, and disease severity.

Significant variation in the type and frequency of podiatry interventions was not an unexpected finding for an exploratory trial with a pragmatic design. However, this approach revealed important factors about the delivery of podiatry care. For example, the definition of the start of treatment was imprecise since patients frequently received an initial assessment consultation and then further appointments during the work up to a physical intervention such as customised orthoses. Notwithstanding the potential non-specific benefits from these consultations, the main physical treatment was often started some months later from baseline assessment. In the present study we also underestimated the 'gatekeeper' role of podiatrist as the rates of referral to other experts was much higher than expected. The extent to which these findings represents local practice or are generalisable to secondary care provision in the UK is unclear. However, with a lack of care pathways and published clinical guidelines further modelling, as suggested in the preclinical and phase I stages of the MRC framework, is required in other centres to define more precisely the constituent parts of podiatry care as well as the extent of other medical, allied health, surgical and self-care involvement. This potential lack of standardisation represents a significant challenge should a definitive trial remain pragmatic and expanded to multi-centre design.

The LFIS provides us with a well-validated patient-focused outcome for use in this type of trial [19]. In an exploratory analysis we detected no change in foot-related impairment and disability and slight deterioration in impairment in the intervention and no interventions groups respectively. This was observed against a backdrop of stable, well-controlled disease. The study was not adequately powered for this outcome but the data generated here and in other pilot studies in our unit will beneficially inform a robust sample size calculation for a definitive trial. For example, based on (previously unreported) data from 141 cases for the primary outcome of LFIS_IF_, a minimally clinically important difference would be 3 points with a standard deviation of 5 points. For a two treatment parallel-design RCT of podiatry-led foot care versus no foot care, 85 per group would be required to detect a difference between the groups of 3 point, based on 90% power and 1% significance level. According to figures from this pilot, a 20% loss to follow-up would be expected so a total sample size of 102 per arm would be required.

Embedded within in a definitive trial should be a qualitative study appraising patient's perceptions and beliefs about their foot care experiences. Efficacy and side-effects are important factors in the evolving experience of RA patients treated pharmacologically, for example with DMARD therapy [22]. Our focus group work revealed low expectation and lack of outcome may have been influenced by poor understanding of the role of podiatry; prior experience from home or over-the-counter remedies such as insoles, foot spas and creams; the inconvenience of additional hospital visits; and lack of benefit gained over the 12 month treatment period. Referring to the MRC framework, the findings from RheumAFooT suggest that further phase I modelling is required as well as refinement to the design of a definitive trial.

## Conclusion

There are increasing calls for foot care to be made more widely available to patients with RA and these are appearing in important clinical guidelines and reports [7,8,12,15,2]. However, podiatry-led care is a complex and complicated intervention and no evidence exists to support its use in these patients. Based on the MRC framework, this exploratory trial has provided useful data to guide the development and implementation of a phase III definitive RCT.

## Competing interests

The author(s) declare that they have no competing interests.

## Authors' contributions

DET conceived of the study, planned the protocol, organised and conducted patient focus groups, and undertook recruitment and clinical data collection

PSH conceived of the study and secured funding, participated in the planning of the study and provided expert advice during data collection and analysis.

JW conceived of the study and secured funding, coordinated the ethical approval, coordinated the trial management group, planned the protocol and conducted the analyses.

All authors read and approved the final manuscript

## Pre-publication history

The pre-publication history for this paper can be accessed here:



## References

[B1] Scott DL (2007). Early rheumatoid arthritis. Br Med Bull.

[B2] Williams AE, Bowden AP (2004). Meeting the challenge for foot health in rheumatic diseases. The Foot.

[B3] Matricali GA, Boonen A, Verduyckt J, Taelman V, Vershueren P, Sileghem A, Corluy L, Westhovens R (2006). The presence of forefoot problems and the role of surgery in patients with rheumatoid arthritis. Ann Rheum Dis.

[B4] Minaker K, Little H (1973). Painful feet in rheumatoid arthritis. Can Med Assoc J.

[B5] Turner DE, Helliwell PS, Emery P, Woodburn J The impact of rheumatoid arthritis on foot function in the early stages of disease: a clinical case series. BMC Musculoskelet Disord.

[B6] Wickman AM, Pinzur MS, Kadanoff R, Juknelis D (2004). Health-related quality of life for patients with rheumatoid arthritis foot involvement. Foot Ankle Int.

[B7] Korda J, Balint GP (2004). When to consult the podiatrist. Best Pract Res Clin Rheumatol.

[B8] Gossec L, Pavy S, Pharm T, Constantin A, Poiraudeau S, Combe B, Flipo RM, Goupille P, Le Loet X, Mariette X, Puechal X, Wendling D, Schaeverbeke T, Sibilia J, Tebib J, Cantagrel A, Dougados M (2006). Nonpharmacological treatments in early rheumatoid arthritis: clinical practice guidelines based on published evidence and expert opinion. Joint Bone Spine.

[B9] Chalmers AC, Busby C, Goyert J, Porter B, Schlzer M (2000). Metatarsalgia and rheumatoid arthritis: a randomised single-blind, sequential trial comparing 2 types of foot orthoses and supportive shoes. J Rheumatol.

[B10] Williams AE, Rome K, Nester CJ (2007). a clinical trial of specialist footwear for patients with rheumatoid arthritis. Rheumatology.

[B11] Davys HJ, Turner DE, Helliwell PS, Conaghan PG, Emery P, Woodburn J (2005). Debridement of plantar callosities in rheumatoid arthritis: a randomized controlled trial. Rheumatology.

[B12] Redmond AC, Waxman R, Helliwell PS (2006). Provision of foot health services in rheumatology. Rheumatology.

[B13] Martin LJ, Griffith SM (2006). High disease activity scores predict the need for additional health services in patients over 60 with rheumatoid arthritis. Musculoskelet Care.

[B14] Farrow SJ, Kingsley GH, Scott DL (2005). Interventions for foot disease in rheumatoid arthritis: a systematic review. Arthritis Rheum.

[B15] Woodburn J, Helliwell P (1997). Foot problems in rheumatology. Br J Rheumatol.

[B16] Medical Research Council (2000). A framework for development and evaluation of RCTs for complex interventions to improve health.

[B17] Arnett FC, Edworthy SM, Bloch DA, Mcshane DJ, Fries JF, Cooper NS, Healey LA, Kaplan SR, Liang MH, Luthra HS, Medsger TA, Mitchell DM, Neustadt DH, Pinals RS, Schaller JG, Sharp JT, Wilder RL, Hunder GS (1988). The American Rheumatism Association 1987 revised criteria for the classification of rheumatoid arthritis. Arthritis Rheum.

[B18] Helliwell PS, Allen N, Gilworth G, Redmond A, Slade A, Tennant A, Woodburn J (2005). Development of a foot impact scale for rheumatoid arthritis. Arthritis Rheum.

[B19] Ward E, King M, Lloyd M, Bower P, Friedli K (1999). Conducting randomized trials in general practice: methodological and practical issues. Br J Gen Pract.

[B20] Altman DG, Bland JM (2005). Treatment allocation by minimisation. BMJ.

[B21] Goodacre LJ, Goodacre JA (2004). Factors influencing the beliefs of patients with rheumatoid arthritis regarding disease-modifying medication. Rheumatology.

[B22] Scottish Intercollegiate Guidelines Network (SIGN) (2000). Management of Early Rheumatoid Arthritis. A National Clinical Guideline. SIGN publication No 48.

